# The Contribution of Botanical Origin to the Physicochemical and Antioxidant Properties of Algerian Honeys

**DOI:** 10.3390/foods13040573

**Published:** 2024-02-14

**Authors:** Sonia Harbane, Olga Escuredo, Yasmine Saker, Asma Ghorab, Rifka Nakib, María Shantal Rodríguez-Flores, Akli Ouelhadj, María Carmen Seijo

**Affiliations:** 1 Ecology, Biotechnology and Health Laboratory, Faculty of Biological Sciences and Agronomic Sciences, University of Mouloud Mammeri, Tizi-Ouzou 15000, Algeria; harbane.sonia@fsbsa.ummto.dz (S.H.); saker.yasmine@fsbsa.ummto.dz (Y.S.); akli.ouelhadj@ummto.dz (A.O.); 2 Faculty of Sciences, University of Vigo, 32004 Ourense, Spain; asma.ghorab@uvigo.gal (A.G.); nakib.rifka@gmail.com (R.N.); mariasharodriguez@uvigo.es (M.S.R.-F.); mcoello@uvigo.es (M.C.S.)

**Keywords:** honey, melissopalynology, physicochemical properties, antioxidant activity, multivariate statistical approach, bioclimatic areas

## Abstract

Honeys from different regions of Algeria were analyzed to determine their pollen characteristics and physicochemical properties (humidity, pH, electrical conductivity, diastase content, color, phenols, flavonoids and antioxidant activity). The antioxidant activity was investigated using the free radical scavenging and Ferric reducing/antioxidant power assays. The melissopalynological analysis revealed 129 pollen types from 53 botanical families. The pollen types found as dominant were *Coriandrum*, *Bupleurum*, *Brassica napus* type, *Hedysarum coronarium*, *Ceratonia siliqua*, *Eucalyptus*, *Peganum harmala*, *Ziziphus lotus* and *Tamarix*. Principal component analysis and cluster analysis were used to analyze significant relationships between the physicochemical variables and the botanical origin of the honeys and establish groupings based on the similarities of their physicochemical and antioxidant properties. The results showed that *Ceratonia siliqua*, *Eucalyptus*, *Arbutus* and honeydew honeys had a higher antioxidant contribution and higher phenolic and flavonoid contents than the rest of the honeys. In addition, the contributions of Mediterranean vegetation such as *Myrtus* and *Phyllirea angustifolia* were significant in this honey group. This paper demonstrates the diverse botanical variability for honey production in Algeria. However, there is a gap in its characterization based on its botanical origin. Therefore, these studies contribute positively to the needs of the beekeeping sector and the commercial valorization of the country’s honey.

## 1. Introduction

Honey is a natural, sweet and flavorful product used since ancient times in different countries due to its functional and therapeutic effects [1]. This bee product contains more than 200 natural substances, mostly composed of sugar and other minor components including organic acids, proteins, enzymes, amino acids, volatile compounds, minerals, flavonoids and phenolic acids [2]. However, its composition can be affected by several factors, such as botanical and geographical origin, climatic conditions, harvest period and storage management [3].

Beekeepers demand the proper identification of the botanical origin of honey and the characteristics that define the particularities of the product for correct authentication. Consequently, they benefit by valuing the bee product with a view to its commercialization. Melissopalynology is one of the best options to provide certainty about the botanical origin of honey. Traditional beekeepers determine the honey type based on the time of nectar appearance and the availability of individual nectar flows. Unifloral honey contains predominantly nectar from a single species and polyfloral honey is produced from the nectar of several plant species, but with none of them being prevalent. However, the success of the identification of geographical and botanical origin of the honey is due to the combination of melissopalynological analysis and physicochemical parameters [4,5,6,7,8]. Until now, some unifloral honeys from Algeria were discriminated based on their pollen profile and physicochemical properties: *Hedysarum coronarium*, *Euphorbia bupleuroides*, *Citrus*, *Retama sphaerocarpa*, *Atractylis*, *Eruca sativa*, *Eucalyptus*, *Brassica*, *Foeniculum*, *Tamarix*, *Asteraceae*, *Capparis*, *Arbutus* and *Ziziphus lotus* [5,6,7,8,9,10,11,12,13]. Nevertheless, a limited number of examined honey samples are found in some published studies. This is a serious issue because the characterization of this product based on its botanical origin will be more accurate the more details there are about the honey’s source, proper handling and conservation by experts, and a sufficient number of samples to examine. The findings and insights offered in this work support the usefulness of Algerian honey, aid in its characterization, and provide details on its potential uses as a functional food and source of medicinal qualities. In the Algerian territory, the vegetation communities are heterogeneous due to the climatic diversity, the soil and the geographical location [11], which reinforces the potential for the production of unifloral honeys. Nectar contributions from specific species can define the particular sensory properties of honey. In this context, there is a need to identify and characterize the physicochemical characteristics and antioxidant properties of honeys depending on the geographical origin, and the specific footprint of this beekeeping product is defined by its botanical origin.

In view of the increasing risk factors to humans for various fatal diseases, there is a global trend towards the study of therapeutic antioxidants. It has been reported that there is an inverse relationship between the dietary intake of antioxidant-rich food and medicinal plants and the incidence of human diseases [1,14]. Many antioxidant compounds naturally occurring in plant sources have been identified as free radical or active oxygen scavengers [14,15,16]. Thus, natural foods obtained from plants such as bee products could be a good therapeutic alternative. Multiple methods have been used to determine the antioxidant activities of honey, including those in vitro (chemical methods and cell models) and in vivo (animal models) [1,17]. The richness of antioxidant substances in honey is due to both enzymatic (e.g., glucose oxidase and catalase) and non-enzymatic components (such as flavonoids, phenolic acids, organic acids, ascorbic acid, amino acids, proteins, Maillard reaction products and carotenoids) [3,14]. However, the antioxidants in honey most studied in relation to their biological activity were polyphenols, mostly flavonoids and simple phenolic derivatives, such as phenolic acids [3,16,18,19]. These chemical substances of honey can neutralize the free radicals by various mechanisms. Different methods are used to assess antioxidant capacity, such as the measurement of the free radical scavenging activity by DPPH assay (2,2-diphenyl-1-picrylhydrazyl) or ABTS assay (2,20-azino-bis (3-ethylbenzothiazoline-6-sulfonic acid), the measurement of the ferric reducing/antioxidant power (FRAP) and oxygen radical absorbance capacity (ORAC) [1,17,20]. Plant polyphenols are considered primary natural antioxidants [18]. Polyphenols have also been reported to affect the physical appearance of honey, particularly honey color. The positive correlation between antioxidant activity, total polyphenols, flavonoid content and honey color has been demonstrated, resulting in dark honeys containing a higher level of phenolic compounds and antioxidant activity [21,22]. Botanical origin and nectar composition provide particular nuances to the color of honey enhancing the dependence between antioxidant activity and botanical origin [3].

In Algeria, the large diversity of vegetation allows for a diversification of honey production [11,12,23]. However, an important part of honey production in this country is still unknown. Algerians are considered traditional consumers of honey, but national production does not achieve self-sufficiency, so to cover the relevant issues, large quantities of honey are imported every year from countries such as China, India and Saudi Arabia [10,11]. In addition, honey consumption has been increasing in recently, due to its recognized healthy properties and different biological activities (such as being antioxidant, antifungal, antibacterial, antiviral, antidiabetic, anti-inflammatory and anticancer) [1]. This has had an impact on the increase in the price of some honey types. As a result, this expensive food product has become the target of dishonest individuals and manufacturers, who profit from this lucrative trade by incorporating cheaper sweeteners into natural honey [24,25]. These fraudulent practices require adequate standards and regulations to guarantee the identity and quality of this bee product [26]. One of the important challenges in terms of quality and food security is the determination of botanical origin because many properties of honey as a food depend on phytochemicals and thus on botanical origin.

Currently, there is a demand from the Algerian beekeeping sector to provide knowledge of its healthy and therapeutic properties based on the botanical origin of the honey produced in each region. Honey is a product consumed naturally, but it can have other industrial uses. Therefore, these scientific contributions provide valuable information about the physicochemical and botanical properties of honey. This fact has a positive impact on its therapeutic potential sought in many functional foods, as an additional ingredient or in the pharmaceutical industry. The main objective of the present study is to determine the botanical origin, the main physicochemical characteristics, color, phenolic and flavonoid contents, and antioxidant activity of Algerian honeys. The influence of the botanical and geographical origins on honey characteristics were analyzed by multivariate techniques. Currently, there is no designation of origin for locally produced honey. This study aims to provide knowledge of specific properties of Algerian honey based on botanical origin, contributing to the valorization of honey production in this country.

## 2. Materials and Methods

### 2.1. The Geographical Origin of the Honey Samples

**Figure 1:**
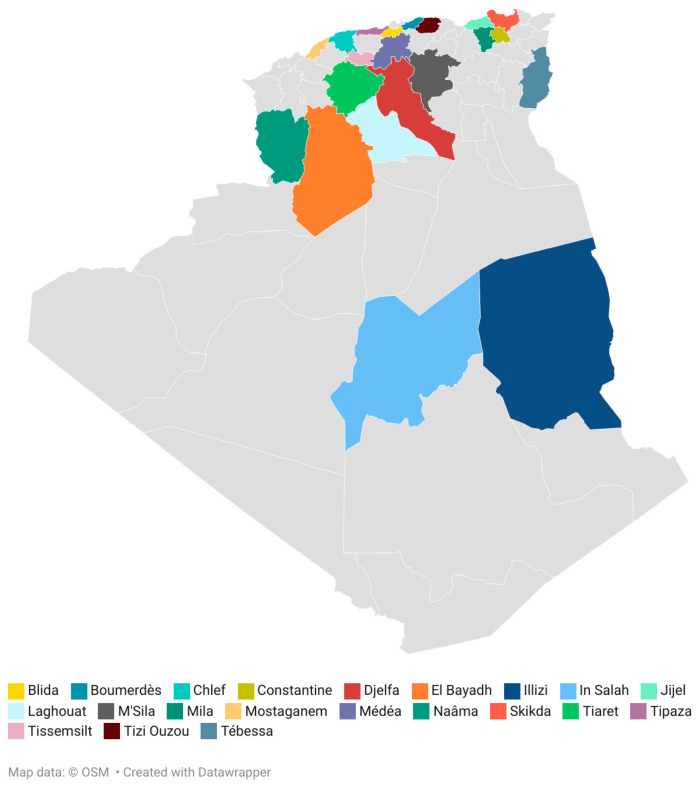
Location of the honey samples. Map created with Datawrapper.

Honey samples obtained from *Apis mellifera* (N = 39) were collected directly from beekeepers (April 2022 to March 2023) in 21 different regions from Algeria. The map shows the location of the Algerian honeys analyzed (Figure 1). The geographical origin of the samples has been differentiated in arid, semi-arid and Mediterranean regions due to the predominant climate of the production area (Table 1). All the obtained honey samples were stored at 4 °C until analyses were performed.

### 2.2. Melissopalynological Analyses

Melissopalynological analyses were carried out using the method described by Louveaux et al. [27], with some modifications [28], based on quantitative and qualitative analysis. For the quantitative analysis, five grams of honey was weighed and completely dissolved in 30 mL of distillated water. Then, the solution was centrifuged for 10 min at 4500 rpm. Afterwards, the supernatant was discarded, and 30 mL of distillated water was added to the sediment and centrifuged again for 5 min at 4500 rpm. The supernatant was discarded again until 5 mL and the sediment was vortexed. Using a micropipette, two drops (10 µL) of the sediment were deposed separately over the slide. The total number of pollen grains in each drop was counted using a light microscope and the result was expressed as the number of pollen grains per 10 g of honey considering the mean value of both drops.

For the qualitative analysis, 30 mL of distillated water was added to the obtained sediment of the first step and the solution was centrifuged in the same condition for 5 min. The supernatant was discarded and the sediment was vortexed. For the microscopic analysis, two drops (100 µL) of the sediment were placed separately in a slide and covered with a cover slide. The pollen spectrum of the sample and the relative frequency of each pollen type (%) was calculated, counting a minimum of 500 pollen grains in the slides. The results were expressed as the relative percentage of each pollen type in each sample and their corresponding frequency classes (P, R, I, A and D): P, a percentage of the pollen type lower than 1%; R, percentage comprising between 1% and 3%; I, percentage between 3% and 15%; A, percentage between 15% and 45%; and D, percentage higher than 45%.

### 2.3. The Determination of the Main Physicochemical Parameters and Color

The physicochemical parameters of honey such as humidity, electrical conductivity, pH and diastase content were performed to confirm the honey quality, according to the harmonized methods of the International Honey Commission [29]. All measurements of physicochemical parameters were performed in duplicate.

Honey color was measured throughout the Pfund scale and CIELab coordinates. A HANNA honey colorimeter (HANNA C221 Honey Color Analyzer, Woonsocket, RI, USA) previously calibrated with glycerol was used to obtain the value in millimeters for the Pfund scale. The CIELab tristimulus determination was performed using a Minolta CR-210 Chroma Meter (Konica Minolta, Tokyo, Japan) previously calibrated with a plate for color coordinates (Υ = 85.8, x = 0.3192, y = 0.3369). The method is based on a sequential or continuous Cartesian representation with three orthogonal axes: L, representing lightness (L = 0, black, and L = 100, colorless), a* green/red color component (a* > 0, red, and a* < 0, green), and b* blue/yellow color component (b* > 0, yellow, and b* < 0, blue).

### 2.4. The Determination of Total Phenolic and Flavonoid Contents

Total phenolic content was determined using Folin–Ciocalteu method [30]. A total of 10 g of honey was dissolved in 30 mL of distillated water (with a concentration of the solution of 0.33 g/mL). Thereafter, the solution was diluted to achieve a 0.11 g/mL. A mixture reactional was prepared with 1 mL of the diluted solution, 10 mL of distillated water and 1 mL of Folin–Ciocalteu. After 2 min, 4 mL of sodium carbonate (7%) was added to the mixture and the volume was completed until 25 mL with distillated water. Finally, the mixture was incubated in the dark for 1 h and the absorbance was measured at 765 nm using UV-VIS spectrophotometer. A calibration curve was obtained using different concentrations of gallic acid solution (0.01–0.50 mg/mL). The results were expressed in gallic acid equivalents in mg/100 g of honey.

Total flavonoid content was determined using a method described by Arvouet-Grand et al. [31], with some modifications. A total of 10 g of honey was diluted in 30 mL of distillated water. A total of 0.5 mL of aluminum chloride (AlCl_3_) was added to 2 mL of honey solution (0.33 g/mL), and the volume was completed with distillated water until it reached 25 mL. The absorbance was measured after incubation for 30 min in the dark using an UV-VIS spectrophotometer at 425 nm. A calibration curve was obtained using different concentrations of quercetin solution (0.002–0.01 mg/mL) as the reference standard. The results were expressed in mg quercetin equivalents per 100 g of honey.

### 2.5. The Determination of Antioxidant Activity

#### 2.5.1. Free Radical Scavenging Activity (DPPH)

The antioxidant activity of honey samples was determined by the discoloration of the DPPH method described by Brand-Williams et al. [32], with some modifications. A total of 5 g of honey sample was dissolved in 50 mL of methanol (0.1 g/mL). Then, 2.7 mL of DPPH solution (0.00006 M) was added to 0.3 mL of the honey solution. A blank was prepared with 0.3 mL of distillated water and 2.7 mL of DPPH solution. The absorbance was measured at 517 nm after incubation in the dark for 30 min. The discoloration of DPPH in each sample tested was calculated by the percentage of radical scavenging activity using the following formula:

DPPH = (A Control − A Sample/A Control) × 100

#### 2.5.2. ABTS Radical Scavenging Assay

The ABTS [2,2′-azino-bis (3-ethylbenzothiazoline-6-sulfonic acid)] radical scavenging activity was assessed by spectrophotometric method [33], with some modifications. Briefly, ABTS solution (7 Mm) was prepared by combining ABTS with potassium persulfate (2.45 mM). The mixture was kept in the dark at room temperature for 12–16 h before use. A total of 1.96 mL of ABTS solution was added to 40 µL of honey solution (0.1 g/mL), then the reactional mixture was incubated in the dark at room temperature for 30 min. The absorbance was measured at 734 nm. The ABTS activity was expressed as a percentage using the following formula:

ABTS scavenging activity = (A Control − A Sample/A Control) × 100

#### 2.5.3. Ferric Reducing/Antioxidant Power Assay (FRAP Assay)

The FRAP test is based on the ability to reduce ferrous iron Fe^3+^ to ferric iron Fe^2+^ using antioxidants [21]. FRAP method was performed according to the methodology described by the authors of [34], with some modifications. The FRAP reagent was prepared by mixing 10 mL of Sodium acetate trihydrate (300 mM), 1 mL of 10 mM TPTZ solution and 1 mL of 20 mM FeCl_3_. A total of 300 µL of honey solution (0.05 g/mL) was added to 2.7 mL of FRAP solution and the absorbance was read at 593 nm after it was incubated at 37 °C for 6 min. A calibration curve was prepared using Trolox as a reference and the results were expressed as µmol of Trolox per g of honey.

### 2.6. Statistical Analyses

Some statistical multivariate treatments such as principal component analysis (PCA) and cluster analysis were applied. First, PCA with the objective of providing a reduced interpretation of the variance of the data was performed. The variables introduced in the statistical analyses were humidity, pH, EC, diastase content, color, total phenols, total flavonoids, antioxidant activity (DPPH, ABTS and FRAP) and the main families and pollen types identified by melissopalynological analyses of honeys. Cluster analysis was carried out to study the similarities and differences in the physicochemical characteristics and pollen profile between the types of honey. The obtained groups were shown graphically based on the botanical origin of the honey and the geographical origin (two areas: arid–semi-arid and Mediterranean). ANOVA test was performed to analyze significant statistical differences between the groups of samples extracted by the cluster analysis. The Bonferroni test was used (*p* < 0.05). These statistical treatments were run using Statgraphics Centurion V18 (Statgraphics Technologies, Inc., The Plains, Fauquier County, VA, USA) and IBM^®^ SPSS^®^ Statistics^®^ version 13.

## 3. Results and Discussion

### 3.1. The Botanical Origin of Honey Samples

**Figure 2:**
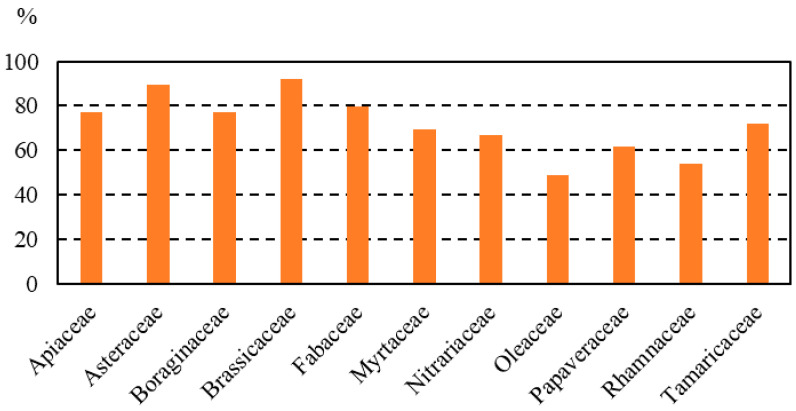
Most representative families in the honey samples analyzed.

The pollen spectra of honey samples showed high diversity in plants. Qualitative melissopalynological analysis revealed 129 pollen types from 53 families. Families found in more than 50% of samples were Brassicaceae (in 92.3% of the honey samples), Asteraceae (89.7%), Fabaceae (79.5%), Apiaceae (76.9%), Boraginaceae (76.9%), Tamaricaceae (71.8%), Myrtaceae (69.2%), Nitrariaceae (66.7%), Papaveraceae (61.5%), Rhamnaceae (53.8%) and Oleaceae (50%) (Figure 2).

Table 2 shows the frequency classes (P, R, I, A, D) of the pollen types identified. The pollen types found to be the dominant pollen (D) were *Coriandrum*, *Bupleurum* (from Apiaceae family), *Brassica napus*-type (from Brassicaceae), *Hedysarum coronarium*, *Ceratonia siliqua* (from Fabaceae), *Eucalyptus* (Myrtaceae), *Peganum harmala* (Nitrariaceae), *Ziziphus lotus* (Rhamnaceae) and *Tamarix* (Tamaricaceae). The secondary pollen types (A) were *Daucus carota* type, *Echium*, *Capparis spinosa*, *Genista* type, *Myrtus* and *Olea europaea*. Important pollen types (I) were *Eryngium campestre* type, *Centaurea* type, *Atractylis* and *Anthemis* type. Some of the pollen types identified are from non-nectariferous plants such as those from families Fagaceae (*Quercus*), Poaceae, Cistaceae or Oleaceae but they provide an interesting data about the plant communities used by honeybees to produce honey. In general, Myrtaceae, Apiaceae, Ericaceae [9,21], Fabaceae and Asteraceae [7,11] are most frequently found families in honey samples from the North of Algeria.

Quantitative analysis showed that most of the samples (27 samples) belong to class II of Maurizio (had pollen contents between 20,000 and 100,000 pollen grains/10 g of honey), followed by 6 samples with pollen contents between 100,000 and 500,000 pollen grains/10 g of honey (class III of Maurizio), 5 samples with a pollen content of less than 20,000 pollen grains/10 g of honey (class I of Maurizio) and one sample belonging to class IV, with between 500,000 and 1,000,000 pollen grains/10 g of honey. This variation in pollen content can be due to the type of honey and other factors such as the hive management and honey extraction, but also the filtering system or the type of hive [7].

### 3.2. Physicochemical Parameters and Color

The average results of the physicochemical parameters analyzed in the 39 honeys are shown in Table 3 and Table S1. The values for humidity ranged between 13.0 and 23.0%, with an average of 17.1%. The highest value exceeded the limit (≤20%) fixed by the Codex Alimentarius standard [35]; however, it corresponds to the sample collected in a humid region (Mediterranean climate) that is from Arbutus, commonly known for its high humidity content. The mean value of pH in the analyzed honeys was 4.1, and ranged between 5.1 (samples collected in the Arid–semi-arid region) and 3.5 (samples from the Mediterranean area). Electrical conductivity (EC) showed a great variation between the analyzed honey samples, ranging from 0.104 to 0.743 mS/cm. These values are below the maximum value of 0.8 mS/cm defined for blossom honey in the Codex Alimentarius (2001) and EU Directive for honey (2002) [35]. The mean diastase content was of 26.3, ranging between 6.0 and 58.1. The results for the physicochemical parameters were similar to those reported for other honeys from Algeria [10,11,21], Turkey [4], Morocco [36,37,38] and Tunisia [39]. However, lower values in Algerian honeys from the Babors Kabylia’s region were reported for diastase content [11].

In the present work, the color of the honey samples was measured using Pfund (mm) and the CIELab system (Table 3). According to Pfund’s index, the color values ranged from 22.5 to 149 mm (equivalent of white to dark), with an average of 66.4 mm. Similar results were found in honeys collected in different biogeographical areas of Algeria [10] and honeys from Tenerife (Spain) [40]. Moreover, the color of Algerian honeys harvested in the Babors Kabylia’s region ranged between light amber and dark amber [11].

The results obtained by the CIELab system are expressed in three coordinates: L, a* and b* (Table 3). The L coordinate, which indicates honey lightness or darkness, ranged from 71.2 (samples from Arid–semi-arid regions) to 97.4 (samples collected in the Mediterranean region). The a* coordinate (redness/greenness) and b* coordinate (blueness/yellowness) were in the ranges of −7.3 to 17.1 and −2.2 to 40.4, respectively. In addition, positive a* indicates red, negative a* indicates green, positive b* indicates yellow, and negative b* indicates blue. The value of L (greater than 50) reflected the light tone of the honeys was analyzed. Moreover, most of the samples had green and yellow colors with negative a* and positive b*, respectively. Similar values in the three coordinates of the CIELab scale measured in honeys from Algeria were reported [6]. However, the lightness values (L) of the Tunisian and Estonian honeys ranged from 36.64 to 51.37 [39] and 65.3 to 90.4 for L [3], respectively, while positive values for a* and b* coordinates in both honey types were found [3,39].

### 3.3. Total Phenolic and Flavonoid Contents and Antioxidant Activity

The concentration of polyphenols varied between 19.7 and 464.1 mg GAE/100 g with a mean of 81.8 mg GAE/100 g (Table 3). These results in polyphenols are superior to other Algerian honeys (20 and 141 mg GAE/100 g) [6,10], Tunisian honeys (16 and 120 mg GAE/100 g) [39], and Estonian honeys (26 and 89 mg GAE/100 g) [3]. Regarding flavonoid content, the mean value obtained for all the samples was 3.1 mg QE/100 g, with maximum and minimum concentrations of 11.0 and 0.3 mg QE/100 g, respectively. In Estonian honeys, similar flavonoid concentrations were found [3] (1.9 to 6.4 mg QE/100 g), but were lower than those of Algerian honeys (3 to 28 mg QE/100 g) [2].

Honey serves as a source of natural antioxidants, which plays an important role in food preservation and human health by combating damage caused by oxidizing agents [20]. Due to the natural antioxidant activity of honey derived from multiple groups of antioxidants, there is no a standardized and ideal method to determine the antioxidant activity of honey. For this reason, the antioxidant activity of the honey samples was determined by three standard spectrophotometric methods: the DPPH and ABTS tests for radical scavenging activity and the FRAP assay for reducing antioxidant power. The percentage of DPPH inhibition in the analyzed honeys varied from 3.0 to 87.1%, with an average of 24.4% and standard deviation of 20.1% (Table 3). These results were closer to those reported by Zaidi et al. [41] for Algerian honeys, with a range between 4.4 to 84%. The percentage of ABTS radical scavenging varied from 3.9 to 51.7%, with mean value of 18.8%. This variation can be attributed to the botanical origins and the presence of various antioxidants (flavonoids, phenolic acids and vitamins C and E) [2]. In the study reported by Zaidi et al. [41], the anti-radical activity of Algerian honey was similar (2.5–63%). It should be noted that the honeys with the greatest radical scavenging power were honeydew and Arbutus honeys, with DPPH and ABTS values higher than 64% and 34%, respectively. The values of antioxidant activity of the honey samples using the FRAP test varied from 50.7 to 237.2 µmol Trolox/g, with a mean value of 87.6 µmol Trolox/g.

### 3.4. The Influence of Botanical and Geographical Origins on the Physicochemical Properties of the Analyzed Algerian Honeys through a Multivariate Statistical Approach

Multivariate techniques were successfully used for the classification and differentiation of honey samples based on botanical origin [7,11,20,23,37,42,43,44]. Principal component analysis aims to find a lower dimensional space, maintaining the greatest possible variability of the data structure [45]. The extracted principal components are linear functions of independent variables in the original data set. The unsupervised approach of this type of analysis offers a visualization of the data structure and the identification of possible groups of data without having a previously predefined group [25].

First, a principal component analysis was performed to analyze relationships among the physicochemical variables and main pollens of the honeys (Table 4). In the present study, the variables used for this purpose were the physicochemical variables, the color by the CIELab scale, the phenolic and flavonoid contents, DPPH, ABTS, FRAP and the families and pollen types best represented according to the melissopalynological analysis (Table 4). This dimensionality-reduction method transforms a large set of variables into a smaller one that still contains most of the information in the large set. The two first components explained 43.43% of the variance of the data. The variables with the highest weight in the first component were FRAP, coordinate a*, DPPH, phenolic content, ABTS and *Myrtus* pollen. In the second component were the physicochemical parameters humidity, coordinates b* and L, pH, and pollen variables *Phyllirea angustifolia*, Arbutus and Ziziphus lotus.

**Figure 3:**
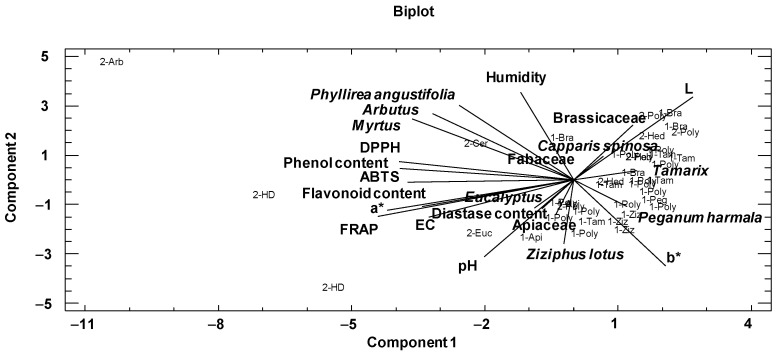
Distribution of pollen and physicochemical variables introduced in the principal component analysis and the graphical representation of honey samples in the first two components. The sample code indicates the botanical origin (Bra: Brassicaceae, Poly: polyfloral, Tam: *Tamarix*, Hed: *Hedysarum coronarium*, Ziz: *Ziziphus lotus*, Peg: *Peganum harmala*, Api: Apiaceae, Euc: *Eucalyptus*, HD: honeydew, Cer: *Ceratonia siliqua*, Arb: *Arbutus*) and geographical area (1: arid and semi-arid areas; 2: Mediterranean areas).

The graphical representation of the first two components is shown in Figure 3. At the left of the plot are situated phenolic and flavonoid contents close to FRAP, DPPH, ABTS, EC and a*coordinate. In addition, *Myrtus*, *Arbutus* and *Phyllirea angustifolia* were situated in the same way; this could be explained by the fact that the samples that contained these pollen types were darker and thus had higher phenol and flavonoid contents and antioxidant activities. At the right, Brassicaceae, *Capparis spinosa*, *Tamarix* and *Peganum harmala* with b* and L coordinates are situated. The samples with these pollen types were light and had lower phenolic and flavonoid contents, and therefore lower antioxidant activities. 

**Figure 4:**
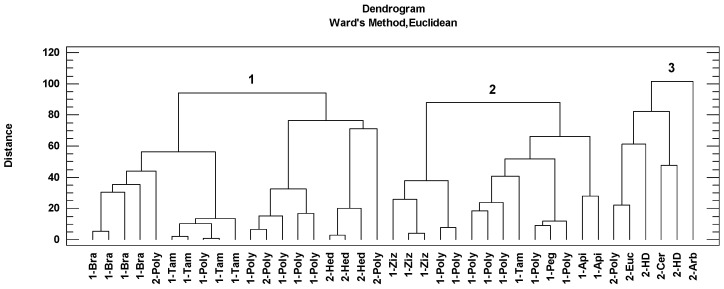
Cluster analysis of the Algerian honey samples based on melissopalynological classification (Bra: Brassicaceae, Poly: polyfloral, Tam: *Tamarix*, Hed: *Hedysarum coronarium*, Ziz: *Ziziphus lotus*, Peg: *Peganum harmala*, Api: Apiaceae, Euc: *Eucalyptus*, HD: honeydew, Cer: *Ceratonia siliqua*, Arb: *Arbutus*) classified the honeys in three groups (1,2 and 3). The number of honey samples indicates arid and semi-arid areas (1) and Mediterranean areas (2).

Cluster analysis is a data reduction technique that facilitates the classification of observations into homogeneous subgroups based on complex and multiple sets of data. This statistical procedure identifies similarities between different cases by grouping them. The grouping of the honey samples according to this analysis is shown in the dendrogram represented by the botanical origin and the geographical origin (Figure 4). Three groups of samples were associated according to the Ward method and Euclidean distance. The first group included nineteen honey samples: four Brassicaceae honeys, four *Tamarix* honeys, three *Hedysarum coronarium* honeys and the rest polyfloral honeys. The second group included fourteen honeys: three *Ziziphus lotus* honeys, one *Tamarix* honey, one *Peganum harmala* honey, two Apiaceae honeys and the rest polyfloral honeys. The third group is composed of one Eucalyptus honey, *Ceratonia siliqua* honey, *Arbutus* honey, two honeydew honeys and one polyfloral honey.

Taking into account geographical origin as a classification variable, it has been possible to group honeys from the Mediterranean climate (cluster 3) (Figure 4). This group of honeys had the higher antioxidant contributions (via the three methods), phenolic and flavonoid contents, significantly higher than the rest of the honeys (Table 5). These honeys also had significantly higher mean values in the main physicochemical parameters (humidity, pH and EC, with mean values of 18.9%, 4.3 and 0.505 mS/cm, respectively). The specific vegetation contributions were *Eucalyptus*, Fabaceae, *Arbutus*, *Myrtus* and *Phyllirea angustifolia*. Higher concentrations of polyphenols in honey with a higher presence of Myrtus and *Hedysarum coronarium* pollens in the Algerian honeys were reported [9]. Therefore, the contribution of these pollens (Mediterranean-vegetation indicators) could contribute favorably to the antioxidant properties of Algerian honeys. In the present study, one sample with a percentage of 74.6% of *Eucalyptus* pollen was found. Many studies reported that *Eucalyptus* was the most observed pollen (as dominant and secondary) found in Algerian honeys [4,46,47]. Myrtaceae is well represented in the studied area due to the presence of *Eucalyptus* (used in reforestation) and Myrtus that grows in Mediterranean areas commonly below 600 m [11]. The importance of these plants for honey production in Mediterranean areas from Algeria were mentioned before [9,11,47]. *Eucalyptus* honey together with Sulla honey are the best known. In the case of this last honey type and according to the palynological analysis, one honey sample was identified with *Hedysarum coronarium* pollen in greater than 85%. Particularly, *Hedysarum coronarium* is widespread in central and northern regions, especially in mountainous areas characterized by herbaceous plant communities dominated by this plant. Other researchers reported this taxon as a dominant or secondary pollen in honey from northeast and central Algeria [9,47].

On the other hand, Cluster 3 included one honey with a percentage of *Arbutus* of 10%. This honey type is classified with a considerably lower percentage of pollen (between 8 and 20%), since the pollen grain is commonly found to be underrepresented [48,49]. Other pollen types identified in *Arbutus* honey were *Quercus* and *Myrtus* (as secondary pollen), and *Brassica napus*-type and *Phyllirea angustifolia* (as minor pollen). This honey sample was collected in Skikda, belonging to the Mediterranean area. It is a rarely studied honey, which in palynological terms has barely been considered [6]. The reason is probably due to the low representation of the pollen grain in the pollen spectrum and the presence of others in a higher amount, which may hide the contribution of this plant to honey production. However, it is a very interesting honey type due to its attributed properties such as having anticancer activities [50]. The literature reported similar results in the physicochemical parameters for *Arbutus* and *Eucalyptus* honeys produced in Algeria [6] and *Arbutus* honeys produced in South Portugal [48]. However, in total, phenol and flavonoid contents and antioxidant capacity were lower than the honeys of this study [48]. In the palynological profile of the two honeydew honeys identified in the present study as main pollens were found *Hedysarum* (Mediterranean areas) and *Ziziphus lotus* (arid–semi-arid areas). Other pollen types such as *Daucus carota*-type, *Pimpinella anisum*-type, *Genista*-type, *Quercus*, *Myrtus* and *Prunus*-type had minor representation in these honeydew honeys. These honeydew honeys differ from blossom honeys (*Hedysarum* and *Ziziphus*) because they had a darker color, greater electrical conductivity, and higher polyphenol content and antioxidant capacity. However, few studies identified honeydew honeys from this geographical origin [10]. These are dark-amber honeys, with values greater than 115 mm on the Pfund scale in comparison with honeydew honeys of other geographical areas [20]. However, honeydew honey produced in other regions such as Spain generally had ECs of around 1 mS/cm [28]. Regarding antioxidant activity, similar values of DPPH inhibition were found in honeydew honey from Poland [20], lower than those of Polish honeydew honeys measured by the FRAP test [51]. In contrast to the honeydew honeys used in this investigation, lower polyphenol concentrations were found in honeydew honeys from Poland [20] and Spain [28].

Statistical analyses revealed that honeys with a greater presence of pollens from the Brassicaceae family, *Capparis spinosa* and *Tamarix* had lower polyphenolic contents and antioxidant activities (grouped in cluster 1) (Table 5). This group included samples from arid and semi-arid regions, but also some honeys from Mediterranean origins (Figure 4). The dominant pollens in these honeys were *Brassica napus*-type (ranging between 57.0 and 69.2%), *Eruca sativa* (50.3 and 74.8%) and *Tamarix* (60.7 and 95.6%). Similar results were found in previous studies of Algerian honeys, with the dominance of *Eruca sativa* [10,12] due to the relevant presence of this species in arid and semi-arid areas. *Brassica napus* was reported as the dominant pollen in honey samples from the M’Sila region in Algeria [10]. With respect to *Tamarix* pollen, a range from 60.7 to 95.6% was found in five samples. These samples had *Eruca sativa* and *Chrysanthemum* type assecondary pollen. Some authors identified *Tamarix* as the dominant pollen in Algerian honeys [10].

Finally, Cluster 2 is formed exclusively by honey samples from arid and semi-arid areas (Figure 4). The honeys of this group were mainly characterized by intermediate values in polyphenols and flavonoids, and antioxidant activity, and with a greater presence of pollens belonging to the Apiaceae family, *Peganum harmala* and *Ziziphus lotus*. *Peganum harmala* was found in one sample as the dominant pollen (62.2%), with *Tamarix* as an accompanying pollen, and *Eucalyptus*, *Eruca sativa*-type and *Centaurea*-type as the minor pollen. Although some papers reported the presence of *Peganum harmala* as a dominant pollen in Algerian honeys [47,52], there is little information on the characterization of this type of honey. The Apiaceae family comprised unifloral honeys, concretely, *Bupleurum* and *Coriandrum* had a representation in the pollen spectra of 56.6% and 87.5%, respectively. Other frequent pollen types in these samples were *Sinapis alba*, *Tamarix* and *Peganum harmala* (although their representation was less than 15%). In recent years, *Ziziphus lotus* honey was the most studied from the physicochemical point of view of the honeys produced in Algeria. According to melissopalynological analysis, *Ziziphus lotus* was found as a dominant pollen in four samples (between 50.3 and 68.1%) in this study. The pollen profile of these honeys contained the pollen types of *Tamarix*, *Eruca sativa*, *Peganum harmala*, *Atractylis* and *Echium* as minor pollen. The jujube (*Ziziphus lotus*) is a deciduous shrub native to the Mediterranean region and the Sahara and differs from other *Ziziphus* species. The plant grows in semi-arid areas and dominates the shrub vegetation. Other authors mentioned *Ziziphus lotus* as a dominant pollen in Algerian honeys and with similar physicochemical characteristics than the honeys analyzed in this paper [8,13,21], whereas other authors [23] reported darker Algerian *Ziziphus* honey samples (103 mm), with higher pH (5.01) and ECs (0.502 mS/cm) than the honeys analyzed in this study. Recently, higher mean values for pH (>4.8) and EC (>1 mS/cm) have been reported in *Ziziphus* honeys from Saudi Arabia and Egypt [53]. It was reported that *Ziziphus* honey in Algeria have higher phenolic and flavonoid contents [8] and also higher DPPH [13]. 

Plant secretions are the primary source of phenolic substances. The concentration and type of phenolic substances in honey varies depending on botanical origin and the geographical location of the floral sources [22]. Therefore, they are the main factors responsible for the antioxidant and biological activities of honey [17,20,21,48,54]. Furthermore, the close relationship with color is due to pigments such as carotenoids and flavonoids, which depend on the botanical and geographical origin of the honey [18]. Hence, the high antioxidant activity found in honeys from *Ceratonia siliqua*, *Eucalyptus*, *Arbutus* and honeydew honeys, with the higher concentration in polyphenols, which agrees with recent studies in honeys from Algeria [6,11]. Moreover, the production of honey in semi-arid areas and arid areas near the Sahara continues to be a pending task. Despite being highly appreciated by consumers, they remain rare and little-known. In collaboration with beekeepers and the country’s administrations, there is much work to be done in the evaluation of the physicochemical characteristics and functional properties of honey produced in Algeria. Consequently, these studies provide valuable information to improve consumer confidence in quality and traceability, thus improving trade in this bee product.

The dependence on multiple variables in the characterization of the botanical origin of honey requires a simultaneous analysis of numerous data. Multivariate statistical techniques are tools that reduce the complexity of large chemical datasets of a food matrix, offering better understanding and precision in the results. This statistical approach has been shown to be successful in the classification of Algerian honey combined with traditional analytical techniques and melisopalynology [11,13,21]. In the future, research with a larger number of samples, with well-identified unifloral pollen profiles related to their physicochemical characteristics linked to this geographical origin will be addressed.

On the other hand, honey has multiple uses in the food industry. In addition to its contribution as a flavor and texture enhancer of many foods, this bee product has important antioxidant activity. Therefore, the honey not only presents itself as a simple food of high nutritional value, but also provides a valuable dietary source of antioxidants. Its recommendation in the diet as an effective food to reduce the risk of developing chronic diseases encourages its use in the food and pharmaceutical industries. The compounds attributable to its healthy properties differ by type of honey (linked to geographical and botanical origin), and therefore their identification contributes to enhancing its physicochemical properties and the valorization of this bee product.

## Figures and Tables

**Table 1:** Geographical origin, climate type, latitude and longitude of honey samples.

Geographical Area	Number of Samples	Climate Type	Latitude	Longitude
Illizi	1	Arid	26°28′59″ N	8°28′00″ E
Constantine	1	Mediterranean	36°21′54″ N	6°36′52″ E
Djelfa	10	Semi-arid	34°40′22″ N	3°15′46″ E
Laghouat	4	Arid	33°47′59″ N	2°51′54″ E
Boumerdès	1	Mediterranean	36°45′37″ N	3°28′20″ E
El-Bayadh	2	Semi-arid	33°40′49″ N	1°01′13″ E
In Salah	1	Arid	27°11′36″ N	2°27′38″ E
Tébessa	1	Semi-arid	35°24′15″ N	8°07′27″ E
M’Sila	4	Semi-arid	35°42′20″ N	4°32′30″ E
Tissemsilt	1	Semi-arid	35°36′25″ N	1°48′38″ E
Médéa	2	Mediterranean	36°15′51″ N	2°45′14″ E
Tiaret	1	Semi-arid	35°22′15″ N	1°19′01″ E
Naâma	1	Semi-arid	33°16′00″ N	0°19′00″O
Blida	1	Mediterranean	36°28′12″ N	2°49′39″ E
Mostaganem	2	Semi-arid	35°55′52″ N	0°05′21″ E
Tipaza	1	Mediterranean	36°35′22″ N	2°26′50″ E
Tizi-Ouzou	1	Mediterranean	36°42′42″ N	4°02′45″ E
Chlef	1	Semi-arid	36°09′54″ N	1°20′04″ E
Jijel	1	Mediterranean	36°49′13″ N	5°46′00″ E
Mila	1	Mediterranean	36°27′01″ N	6°15′51″ E
Skikda	1	Mediterranean	36°52′34″ N	6°54′33″ E

**Table 2:** Relevant families and pollen types identified in the honey samples. P: present pollen (<1%); R: minor pollen (1–3%); I: important pollen (3–15%); A: secondary pollen (15–45%); D: dominant pollen (>45%); t: pollen type.

Family	Pollen Type	P	R	I	A	D
Apiaceae	*Coriandrum*	17.9	-	5.1	-	2.6
	*Bupleurum*	5.1	2.6	-	-	2.6
	*Daucus carota* t	20.5	2.6	2.6	2.6	-
	*Pimpinella anisum* t	5.1	10.3	5.1	-	-
	*Eryngium campestre* t	25.6	7.7	2.6	-	-
	*Ferula communis* t	2.6	2.6	2.6	-	-
Asteraceae	*Centaurea* t	20.5	15.4	20.5	-	-
	*Atractylis*	25.6	10.3	10.3	-	-
	*Anthemis* t	20.5	5.1	10.3	-	-
	*Aster* t	10.3	7.7	2.6	-	-
	*Scorzonera* t	20.5	5.1	2.6	-	-
	*Galactites* t	15.4	2.6	2.6	-	-
	*Arctium* t	7.7	2.6	2.6	-	-
Boraginaceae	*Echium*	25.6	30.8	17.9	2.6	-
Brassicaceae	*Brassica napus* t	5.1	12.8	38.5	25.6	10.3
Capparaceae	*Capparis spinosa*	-	-	-	2.6	-
Ericaceace	*Arbutus*	-	-	2.6	-	-
Euphorbiaceae	*Euphorbia* t	15.4	5.1	2.6	-	-
Fabaceae	*Hedysarum coronarium*	10.3	15.4	2.6	5.1	10.3
	*Ceratonia siliqua*	-	2.6	-	-	2.6
	*Genista* t	17.9	23.1	15.4	5.1	-
	*Astragalus*	2.6	2.6	2.6	-	-
Lythraceae	*Punica granatum*	7.7	-	7.7	-	-
Myrtaceae	*Eucalyptus*	46.2	5.1	7.7	5.1	5.1
	*Myrtus*	7.7	-	5.1	2.6	-
Nitrariaceae	*Peganum harmala*	7.7	12.8	33.3	10.3	2.6
Oleaceae	*Olea europaea*	17.9	20.5	7.7	2.6	-
	*Phyllirea angustifolia*	-	-	5.1	-	-
Papaveraceae	*Papaver rhoeas* t	25.6	20.5	15.4	-	-
Rhamnaceae	*Ziziphus lotus*	15.4	10.3	10.3	7.7	10.3
Rosaceae	*Prunus* t	17.9	5.1	2.6	-	-
Tamaricaceae	*Tamarix*	10.3	2.6	17.9	28.2	12.8

**Table 3:** Physicochemical parameters, phenolic content, flavonoid content and antioxidant activity of analyzed honey samples.

	Mean	SD	Maximum	Minimum
Humidity (%)	17.1	2.4	23.0	13.0
pH	4.1	0.3	5.1	3.5
Electrical conductivity (mS/cm)	0.291	0.165	0.743	0.104
Diastase content	26.3	13.3	58.1	6.0
Color (mm Pfund)	66.4	26.0	149.0	22.5
L	86.0	5.1	97.4	71.2
a*	−2.3	4.9	17.1	−7.3
b*	22.8	9.5	40.4	−2.2
Phenolic content (mg GAE/100 g)	81.8	82.1	464.1	19.7
Flavonoid content (mg QE/100 g)	3.1	2.1	11.0	0.3
DPPH (%)	24.4	20.1	87.1	3.0
ABTS (%)	18.8	12.2	51.7	3.9
FRAP (µmol Trolox/g)	87.6	38.1	237.2	50.7

SD: standard deviation. L represents lightness, a* green/red color component and b* blue/yellow color component according to CIELab scale.

**Table 4:** Number of components extracted and pollen and physicochemical variable weights introduced in the principal component analysis.

	Number of Components							
	1	2	3	4	5	6	7	8
Eigenvalue	6.77	3.22	1.90	1.76	1.35	1.31	1.26	1.04
Variance (%)	29.44	13.98	8.28	7.63	5.88	5.69	5.47	4.50
Cumulative variance (%)	29.44	43.43	51.71	59.34	65.22	70.90	76.38	80.88
	Component weights for each variable							
Physicochemical variables								
Humidity	−0.09	0.37	−0.06	−0.19	0.25	−0.05	−0.18	−0.22
pH	−0.16	−0.32	0.29	−0.01	0.29	0.00	0.15	0.02
Electrical conductivity (EC)	−0.26	−0.16	0.15	−0.16	−0.14	−0.24	−0.14	−0.12
Diastase content	−0.06	−0.12	0.28	0.48	−0.16	−0.03	−0.10	−0.16
L	0.21	0.34	0.07	0.24	0.01	−0.13	0.04	0.00
a*	−0.33	−0.13	−0.16	0.00	0.06	0.05	−0.06	0.22
b*	0.17	−0.36	0.04	−0.24	−0.06	0.09	−0.11	−0.22
Phenol content	−0.31	0.05	−0.19	0.25	−0.06	0.10	0.04	0.24
Flavonoid content	−0.27	−0.11	−0.15	0.20	−0.13	0.25	−0.09	0.16
DPPH	−0.31	0.08	−0.08	0.07	0.18	−0.19	−0.10	0.10
ABTS	−0.30	−0.01	−0.01	0.23	0.15	0.18	−0.04	−0.10
FRAP	−0.35	−0.15	0.01	−0.02	0.16	−0.07	−0.03	−0.03
Pollen variables								
Apiaceae	−0.03	−0.14	−0.17	−0.09	0.26	0.47	0.45	−0.37
*Arbutus*	−0.25	0.28	0.28	−0.19	−0.17	0.09	0.09	−0.07
Brassicaceae	0.11	0.23	0.11	0.25	0.43	0.07	−0.11	0.07
*Capparis spinosa*	0.05	0.10	−0.07	−0.08	0.17	−0.20	0.44	0.50
*Eucalyptus*	−0.07	−0.12	−0.15	−0.32	0.24	−0.31	−0.40	0.03
Fabaceae	−0.05	0.06	−0.51	0.16	−0.41	−0.21	0.04	−0.26
*Myrtus*	−0.29	0.25	0.20	−0.14	−0.20	0.08	0.05	−0.02
*Peganum harmala*	0.10	−0.11	0.07	−0.26	−0.30	0.32	−0.12	0.48
*Phyllirea angustifolia*	−0.20	0.31	0.27	−0.23	−0.14	0.05	0.14	−0.10
*Tamarix*	0.11	0.04	0.24	0.16	0.03	0.30	−0.44	0.06
*Ziziphus lotus*	−0.02	−0.27	0.37	0.18	−0.08	−0.39	0.26	0.01

**Table 5:** Average values of the physicochemical properties and the main pollen families and types identified in the honeys according to the three groups extracted from the cluster analysis. Different letters show significant differences according to Bonferroni test (*p* < 0.05).

	Cluster 1	Cluster 2	Cluster 3
Physicochemical variables			
Humidity (%)	18.1 b	15.0 a	18.9 b
pH	3.9 a	4.2 b	4.3 a
Electrical conductivity (mS/cm)	0.207 b	0.313 b	0.505 a
Diastase content	22.7	32.6	26.5
L	88.8 a	84.0 b	81.4 b
a*	−4.7 b	−2.5 b	6.0 a
b*	21.8	27.0 a	15.8 b
Phenol content (mg GAE/100 g)	49.4 b	74.1 b	202.2 a
Flavonoid content (mg QE/100 g)	2.3 b	3.3	5.4 a
DPPH (%)	18.6 b	14.4 b	65.8 a
ABTS (%)	15.6 b	16.4 b	34.5 a
FRAP (µmol Trolox/g)	67.3 b	84.6 b	158.8 a
Pollen variables (%)			
Apiaceae	1.7 b	14.6 a	1.2 b
*Arbutus*	0	0	1.0
Brassicaceae	23.8 a	7.2 b	3.0 b
*Capparis spinosa*	1.9 a	0 b	0 b
*Eucalyptus*	1.3 b	1.7 b	24.7 a
Fabaceae	16.9	7.8	22.5
*Myrtus*	0 b	0.2 b	4.6 a
*Peganum harmala*	4.3 b	15.1 a	0.1 b
*Phyllirea angustifolia*	0.3	0	1.7
*Tamarix*	23.8	15.6	0.1
*Ziziphus lotus*	2.0 b	18.8 a	9.5

## Data Availability

Data is contained within the article or Supplementary Material.

## Supplementary Materials

The following supporting information can be downloaded at: https://www.mdpi.com/article/10.3390/foods13040573/s1, Table S1: Physicochemical characteristics of each honey sample.
