# Supplementary material for: The Contribution of Botanical Origin to the Physicochemical and Antioxidant Properties of Algerian Honeys
Source: Foods. 2024 Feb 14;13(4):573. doi: 10.3390/foods13040573 (PMC10888090; doi:10.3390/foods13040573)
Supplement: Supplementary file 1 [file foods-13-00573-s001.zip › foods-2859285-supplementary.pdf]

**Table S1**. Physicochemical characteristics of each honey sample.

| Samples | Humidity (%) | pH | EC (mS/cm) | Diastase content | Color (mm Pfund) | L | a* | b* | Phenolic content (mg GAE/100 g) | Flavonoid content (mg QE/100 g) | DPPH (%) | ABTS (%) | FRAP (μmol Trolox /g) |
|---|---|---|---|---|---|---|---|---|---|---|---|---|---|
| 1 | 15.1 | 4.1 | 0.16 | 29.4 | 57.0 | 88.4 | -5.3 | 24.0 | 33.8 | 2.3 | 17.0 | 20.4 | 62.0 |
| 2 | 21.0 | 4.0 | 0.14 | 6.0 | 31.5 | 90.2 | -4.8 | 12.3 | 35.3 | 0.3 | 16.9 | 15.3 | 65.0 |
| 3 | 20.3 | 4.0 | 0.11 | 28.1 | 28.5 | 91.1 | -4.9 | 15.0 | 39.4 | 0.6 | 20.3 | 7.2 | 65.7 |
| 4 | 18.0 | 4.0 | 0.15 | 30.5 | 34.0 | 89.9 | -5.1 | 20.2 | 29.9 | 1.3 | 13.6 | 5.4 | 65.3 |
| 5 | 17.0 | 4.1 | 0.35 | 24.0 | 58.5 | 86.1 | -4.1 | 28.6 | 68.4 | 1.5 | 35.9 | 29.4 | 82.5 |
| 6 | 19.1 | 3.9 | 0.16 | 22.3 | 56.0 | 90.5 | -6.2 | 23.8 | 49.9 | 2.1 | 14.7 | 21.9 | 73.7 |
| 7 | 19.0 | 3.9 | 0.10 | 21.9 | 32.5 | 92.4 | -4.8 | 25.2 | 42.0 | 2.0 | 12.7 | 25.7 | 65.8 |
| 8 | 13.7 | 4.4 | 0.14 | 8.3 | 74.0 | 84.6 | -2.8 | 25.3 | 71.8 | 0.9 | 23.6 | 18.0 | 84.0 |
| 9 | 13.1 | 4.3 | 0.22 | 26.4 | 104.5 | 78.2 | 3.1 | 27.6 | 54.9 | 5.6 | 5.6 | 21.7 | 84.4 |
| 10 | 15.0 | 4.1 | 0.46 | 58.1 | 95.0 | 81.4 | -0.2 | 22.7 | 69.4 | 2.6 | 9.3 | 21.8 | 85.6 |
| 11 | 14.4 | 4.1 | 0.19 | 11.4 | 70.5 | 85.8 | -2.6 | 28.6 | 45.6 | 2.4 | 10.2 | 5.7 | 69.7 |
| 12 | 14.0 | 4.3 | 0.60 | 26.6 | 106.0 | 82.1 | -0.9 | 26.7 | 52.0 | 5.9 | 8.2 | 10.1 | 84.2 |
| 13 | 13.0 | 4.1 | 0.35 | 36.6 | 71.0 | 88.1 | -5.6 | 22.8 | 67.6 | 2.0 | 16.4 | 12.4 | 76.2 |
| 14 | 15.0 | 4.2 | 0.25 | 56.6 | 65.0 | 89.5 | -5.6 | 27.1 | 95.1 | 3.0 | 19.5 | 12.5 | 81.1 |
| 15 | 15.5 | 4.2 | 0.27 | 55.7 | 77.0 | 86.6 | -4.4 | 28.6 | 93.1 | 3.7 | 18.6 | 34.1 | 90.8 |
| 16 | 16.0 | 3.9 | 0.40 | 25.7 | 61.5 | 85.5 | -5.0 | 31.0 | 58.8 | 2.9 | 15.0 | 7.4 | 68.1 |
| 17 | 13.9 | 4.5 | 0.47 | 47.1 | 60.0 | 88.6 | -6.4 | 22.6 | 52.8 | 2.9 | 3.0 | 7.9 | 72.0 |
| 18 | 17.9 | 4.2 | 0.21 | 16.3 | 63.5 | 83.0 | -3.6 | 32.3 | 75.7 | 2.6 | 13.9 | 8.4 | 66.9 |
| 19 | 15.6 | 4.3 | 0.36 | 25.8 | 94.0 | 80.1 | 0.0 | 26.8 | 86.1 | 4.8 | 17.8 | 13.2 | 103.4 |
| 20 | 16.8 | 4.2 | 0.30 | 15.9 | 70.5 | 83.1 | -1.5 | 31.0 | 62.0 | 2.4 | 50.3 | 11.3 | 96.9 |
| 21 | 19.0 | 5.1 | 0.74 | 28.7 | 112.5 | 71.2 | 11.4 | 32.0 | 133.3 | 5.1 | 71.0 | 34.4 | 237.2 |
| 22 | 23.0 | 4.2 | 0.71 | 30.6 | 86.0 | 82.7 | 7.1 | -2.2 | 276.4 | 6.6 | 82.1 | 51.7 | 186.9 |
| 23 | 17.0 | 4.3 | 0.23 | 34.9 | 63.5 | 97.4 | -0.4 | 2.2 | 247.4 | 4.1 | 87.1 | 33.4 | 119.6 |
| 24 | 19.0 | 4.4 | 0.63 | 26.9 | 82.0 | 78.4 | 2.2 | 30.3 | 30.3 | 3.0 | 39.5 | 29.5 | 142.9 |

| | | | | | | | | | | | | | | |
|---|---|---|---|---|---|---|---|---|---|---|---|---|---|---|
| 25 | 16.7 | 3.9 | 0.43 | 22.0 | 149.0 | 75.7 | 17.1 | 1.4 | 464.1 | 11.0 | 64.9 | 46.9 | 169.7 |
| 26 | 18.1 | 3.9 | 0.18 | 17.6 | 80.0 | 80.5 | 0.6 | 30.8 | 126.0 | 3.1 | 26.4 | 18.1 | 103.2 |
| 27 | 15.5 | 4.9 | 0.27 | 21.5 | 91.0 | 82.5 | -2.1 | 25.3 | 88.0 | 4.1 | 14.0 | 38.4 | 114.4 |
| 28 | 20.4 | 3.8 | 0.35 | 23.2 | 61.5 | 88.4 | -5.8 | 24.8 | 50.3 | 6.0 | 19.8 | 5.9 | 64.4 |
| 29 | 16.7 | 3.8 | 0.16 | 32.6 | 64.5 | 86.7 | -7.3 | 40.4 | 43.8 | 4.8 | 18.2 | 27.0 | 75.9 |
| 30 | 15.9 | 3.9 | 0.18 | 32.0 | 48.0 | 85.8 | -3.3 | 27.8 | 58.6 | 2.3 | 22.9 | 24.3 | 76.8 |
| 31 | 18.0 | 4.2 | 0.14 | 48.7 | 66.0 | 91.6 | -0.8 | 1.3 | 144.5 | 5.3 | 33.2 | 31.3 | 80.3 |
| 32 | 16.0 | 4.0 | 0.18 | 25.0 | 51.0 | 87.2 | -5.4 | 30.6 | 58.1 | 3.7 | 20.8 | 17.8 | 71.6 |
| 33 | 20.4 | 3.5 | 0.26 | 11.7 | 34.0 | 88.3 | -5.7 | 23.7 | 46.9 | 0.6 | 17.1 | 14.7 | 59.9 |
| 34 | 17.3 | 3.9 | 0.15 | 33.7 | 73.5 | 84.4 | -2.5 | 30.3 | 58.9 | 3.8 | 13.1 | 20.9 | 71.4 |
| 35 | 21.0 | 3.9 | 0.35 | 21.5 | 64.0 | 87.5 | -3.6 | 20.0 | 38.9 | 2.1 | 21.8 | 5.2 | 63.8 |
| 36 | 17.0 | 3.9 | 0.14 | 10.4 | 37.5 | 90.7 | -4.8 | 15.8 | 41.4 | 1.4 | 17.1 | 6.3 | 52.0 |
| 37 | 17.0 | 3.7 | 0.40 | 8.8 | 50.5 | 89.0 | -5.8 | 19.7 | 39.4 | 2.4 | 16.4 | 7.0 | 70.9 |
| 38 | 16.0 | 3.8 | 0.30 | 11.8 | 43.5 | 89.0 | -4.7 | 18.9 | 39.1 | 1.3 | 15.0 | 6.8 | 60.7 |
| 39 | 19.1 | 4.1 | 0.15 | 9.6 | 22.5 | 90.3 | -5.0 | 12.2 | 19.7 | 0.4 | 7.5 | 3.9 | 50.7 |
